# Tolerance and Persistence of Ebola Virus in Primary Cells from *Mops condylurus*, a Potential Ebola Virus Reservoir

**DOI:** 10.3390/v13112186

**Published:** 2021-10-29

**Authors:** Marcel Bokelmann, Uwe Vogel, Franka Debeljak, Ariane Düx, Silke Riesle-Sbarbaro, Angelika Lander, Annette Wahlbrink, Nicole Kromarek, Stuart Neil, Emmanuel Couacy-Hymann, Joseph Prescott, Andreas Kurth

**Affiliations:** 1 Centre for Biological Threats and Special Pathogens, Robert Koch Institute, 13353 Berlin, Germany; Marcel.Bokelmann@fli.de (M.B.); VogelU@rki.de (U.V.); Riesle-SbarbaroS@rki.de (S.R.-S.); LanderA@rki.de (A.L.); wahlbrinka@rki.de (A.W.); kromarekn@rki.de (N.K.); prescottj@rki.de (J.P.); 2 Department of Infectious Diseases, King’s College London, London WC2R 2LS, UK; franka.debeljak@kcl.ac.uk (F.D.); stuart.neil@kcl.ac.uk (S.N.); 3 Epidemiology of Highly Pathogenic Microorganisms, Robert Koch Institute, 13353 Berlin, Germany; DuexA@rki.de; 4 Laboratoire National d’Appui au Développement Agricole, Bingerville BP 206, Côte d’Ivoire; chymann@gmail.com

**Keywords:** Ebola virus, reservoir host, bat, virus replication, tolerance, persistent infection

## Abstract

Although there have been documented Ebola virus disease outbreaks for more than 40 years, the natural reservoir host has not been identified. Recent studies provide evidence that the Angolan free-tailed bat (*Mops condylurus*), an insectivorous microbat, is a possible ebolavirus reservoir. To investigate the potential role of this bat species in the ecology of ebolaviruses, replication, tolerance, and persistence of Ebola virus (EBOV) were investigated in 10 different primary bat cell isolates from *M. condylurus*. Varying EBOV replication kinetics corresponded to the expression levels of the integral membrane protein NPC1. All primary cells were highly tolerant to EBOV infection without cytopathic effects. The observed persistent EBOV infection for 150 days in lung primary cells, without resultant selective pressure leading to virus mutation, indicate the intrinsic ability of EBOV to persist in this bat species. These results provide further evidence for this bat species to be a likely reservoir of ebolaviruses.

## 1. Introduction

Ebolavirus and Marburgvirus are genera within the family Filoviridae in the order of Mononegavirales [1]. Six species within the Ebolavirus genus have been discovered: *Zaire*, *Sudan*, *Taï Forest*, *Bundibugyo*, *Reston* and, most recently, *Bombali ebolavirus.* Of these six species, only four viruses (Ebola virus, Sudan virus, Taï Forest virus, and Bundibugyo virus) are known to cause severe hemorrhagic fever in humans with case fatality rates up to 90% [1,2,3]. Since 1976, 29 ebolavirus outbreaks have been documented in Africa. The largest outbreak, occurring in 2014–2016, was caused by Ebola virus (EBOV), resulting in over 28,600 cases and 11,300 deaths [4].

Outbreak investigations and several epidemiological studies provide evidence that several bat species are the likely natural reservoir hosts for ebolaviruses [5,6]. For multiple outbreaks, there is anecdotal evidence of index patients contacting bats prior to infection [5,7,8,9,10]. Various species of wild-caught bats have tested seroreactive for EBOV, with antibodies being detected in 307 individual bats from 17 species in Africa and Asia [5,11,12,13,14,15,16,17,18,19,20]. Almost all of these studies have focused on frugivorous bats, while insectivorous microbats have received only sparse attention in ebolavirus research [21]. With EBOV replication without signs of illness after experimental infection [22], detection of EBOV-specific antibodies [13] and a potential contact with the index case of the large EBOV outbreak in West Africa [7], some studies provide evidence that the insectivorous Angolan free-tailed bat (*Mops condylurus*) is a potential reservoir of ebolaviruses. The discovery of a new ebolavirus species, *Bombali ebolavirus*, in *M. condylurus* in Sierra Leone [23] and repeated detection in *M. condylurus* in Kenya [24,25] and Guinea [26] indicates that further investigation into the role that this microbat has in the ecology of ebolaviruses is important. Although *Bombali ebolavirus* has not been isolated from a wild-caught *M. condylurus* bat yet, its role as natural reservoir for this virus is highly probable. In this study, we focus our investigations on the potential role of this microbat in the ecology of EBOV.

A key component in the filovirus entry process is the integral membrane protein Niemann-Pick C1 (NPC1), found in late endosomes and lysosomes [27] and utilized in the viral entry process by the glycoproteins (GP) of Ebola virus [28,29,30], Marburg virus (MARV) [31], and Měnglà virus [32], a recently discovered filovirus infecting fruit bats in China. NPC1 knockout cells are refractory to EBOV infection [27,33]. Additionally, NPC1 was shown to be a genetic determinant of filovirus susceptibility in bat cells; NPC1 polymorphisms found in specific bat species result in reduced interactions between filoviruses and NPC1, influencing the cellular susceptibility of bats to filovirus infection, replication, and virulence [34]. In culture, *M. condylurus* cells typically have low NPC1 expression levels compared to cells from highly symptomatic hosts, such as humans [35]. Infection of *M. condylurus* cells with EBOV revealed a potential correlation between NPC1 receptor expression level and virus replication rate [35].

A critical requirement for the identification of a natural reservoir of a given zoonotic virus is evidence of viral persistence at the population level, which can only be determined through longitudinal studies [36]. When elucidating the hypothetical circulation and maintenance of EBOV in a bat species, high population densities in wild bat colonies and metapopulation structures with bat migration between colonies have to be considered. Virus spread within the colony may be rapid and result in a decrease in the number of susceptible individuals (through induction of protective immunity) to a level too low to maintain virus transmission [37]. As viruses are obligate intracellular parasites that must be maintained in a population, RNA viruses have evolved a number of strategies to counteract the dead-end case of depletion of susceptible individuals [37]. One strategy is the establishment of persistent infections in at least some individuals, who maintain the virus for months, years, or even lifelong and can then act as a periodic source of the virus within a host population [37]. This strategy has been observed for several RNA viruses such as Foot and mouth disease virus (FMDV) [38,39], Borna disease virus (BDV) [40,41,42], Bluetongue virus (BTV) [43,44], Measles morbillivirus (MeV) [45,46,47], Hepatitis C virus (HCV) [48,49,50], and Zika virus (ZIKV) [51,52,53]. Long-term studies with Nipah virus (NiV)-infected *Pteropus vampyrus* and MARV-infected *Rousettus aegyptiacus* fruit bats provide evidence for virus persistence in individuals of these reservoir species [54,55,56] and MARV has been also shown to establish testicular persistence in macaques [57]. EBOV has been shown to persist in immune-privileged sites such as eye, brain, and testes in humans [58,59,60,61] and rhesus monkeys [62], although this is not a main driver of virus transmission. The resurgence of EBOV in 2021 in Guinea from persistently infected humans [63] underlines the importance to investigate filovirus persistence. The fact that humans can become persistently infected may also reflect the intrinsic ability of EBOV to establish persistence in its natural host [37]. The establishment of a persistent infection in a host requires a supply of susceptible cells dividing at a similar pace as the virus replicates, and the ability to hide from, or subvert the host’s immune response [64]. If cells continue to synthesize high levels of viral proteins, cells will likely suffer cytopathic effects, either as a direct consequence of virus replication, or elimination by host immune responses. Therefore, virus replication needs to be repressed in at least some infected cells to a level low enough to avoid the above consequences [37]. Mechanisms of persistence may entail: (1). low level virus replication within cells remaining persistently infected (e.g., BDV), (2). infections in which the virus slowly spreads from cell to cell, but during which the infected cells may die (e.g., rabies virus), or (3). infections in which the virus latently hides without apparent replication, (e.g., BTV in erythrocytes) [37].

The aim of this study was to investigate indicators that elucidate the role of *M. condylurus* as a reservoir and their importance in the ecology of ebolaviruses. For this, we determined the EBOV replication kinetics and assessed viral tolerance and persistence in primary cells from *M. condylurus*. We found that in most cases, lower EBOV replication rates in primary cells from *M. condylurus* corresponded to lower NPC1 receptor expression levels. High tolerance to EBOV infections without cell death and establishment of persistent infection in specific *M. condylurus* cells provided further evidence that this bat species is important in the ecology of ebolaviruses.

## 2. Materials and Methods

### 2.1. Generation of CRISPR Knockout Cell Line (HEK293∆NPC1)

For NPC1 gene knock out, a guide targeting early exon (5′ ACT GAA CCT GTT TTG TGA GC 3′) was designed using the GPP sgRNA Designer from the Broad institute [65,66]. The guide sequence was cloned into a lentiCRISPRv2 plasmid (Addgene, Watertown, MA, USA) as described previously [67], encoding GFP for selection. The plasmid was cut using BsmBI restriction enzyme (New England Biolabs, Ipswich, MA, USA), followed by gel purification (Qiagen Gel Purification Kit, Qiagen, Hilden, Germany). Next, the oligo pairs targeting the gene of interest were annealed and phosphorylated by using T4 PNK (New England Biolabs), before being ligated into the cut lentiCRISPRv2 plasmid. 293TIM1 cells were plated in a 6-well plate (Corning Inc., Corning, NY, USA) and transfected using PEI Max (PEI max, 1 mg/mL in ddH_2_O, Polysciences, Warrington, PA, USA) with 250 ng of lentiCRISPRv2 plasmid encoding the guide of interest and medium was changed (DMEM, high glucose, GlutaMAX™ (Gibco/Thermofisher, Waltham, MA, USA)) supplemented with 10% FCS (GIBCO/Thermofisher) and gentamycin (20 μg/mL—Gibco/Thermofisher) after 6 h. Then, 48 h after transfection, cells were selected and single-cell sorted by flow cytometry (FACS Canto II cytometer, BD Biosciences, Franklin Lakes, NJ, USA) on the basis of their expression level of GFP. The selected cells were then analyzed by Western blot to confirm the loss of expression of the target protein. The genomic DNA from sorted clones was then isolated and CRISPR-generated lesions were confirmed by sequencing.

### 2.2. Cell Cultures

All microbat cell cultures, media, and culture conditions, were described in detail previously by our laboratory [35]. For infection experiments with EBOV, we selected 15 unique cell cultures (Table 1): primary cell isolates from *M. condylurus* (9) and the European bat species *Nyctalus noctula* (1), immortalized cells from *M. condylurus* (1), monkey (1), and human (3). These cells exhibited a wide range of NPC1 receptor expression levels [35]: HEK293∆NPC1 cells absent of NPC1 expression; MoTra Prim, MoLu Prim, MoSp Prim Early, MoKi Prim, and MoSk Prim cells represented low NPC1 expression; MoLi Prim, MoTes Prim, MoBra Prim, and immortalized MoKi cells represented moderate NPC1 expression; MoSp Prim Late, NyKi Prim, Vero, HeLa, and HEK293 cells represented high NPC1 receptor expression levels. Cell viability was determined using an automated cell counter (EVE™, NanoEnTek, Seoul, Korea).

### 2.3. Infections and Viral RNA Quantitation

Infectious work with EBOV and MARV was performed in the BSL4 facility at the Robert Koch Institute (Berlin, Germany), according to standard operating protocols (SOPs). Cells were seeded in 6-well plates and inoculated in triplicates with EBOV (strain Makona, C05; adaptation: 2 passages on MoKi cells) at 3 × 10^5^ TCID_50_/well, EBOV-GFP with MOI 0.1 or MARV (strain Musoke) at 10^4^ TCID_50_/well for 1 h. Cells were then washed twice with PBS, after which 3 mL cell culture medium was added. For EBOV quantitation, 140 µL supernatant per well was collected in AVL Buffer (19073, Qiagen, Hilden, Germany) after 1, 24, 72, and 96 h, or weekly until 150 dpi for persistently infected cells. Samples were then mixed with an equal volume of 100% ethanol before removal from the BSL4. Viral RNA was extracted using the QIAamp Kit (Qiagen) according to the manufacturer’s instructions. Viral RNAs were quantified by qRT-PCR (Applied Biosystems^TM^ 7500, Waltham, MA, USA) using the AgPath-ID^TM^ One-Step RT-PCR Kit (4387391, Thermo Fisher, Waltham, MA, USA). Primers/probe and cycling conditions were described previously [35]. Viral RNA copy numbers of each sample were calculated from a standard curve, that was produced using EBOV in vitro transcripts (concentrations ranging from 10 to 10^7^ copies/µL). Differences in replication rates were calculated as geometric mean of triplicates in lg(x) (viral RNA variation within triplicates did not allow statistical calculations).

### 2.4. Virus Titration

MoKi cells were seeded into 96-well plates at 5 × 10^4^ cells in 100 μL titration medium (DMEM containing 10% FCS, 2 mM glutamine, and 1 × penicillin/streptomycin) for virus titration on the following day. Supernatants from infected cell cultures were serially diluted from 10^−1^ to 10^−9^ in titration medium containing 5% FCS. Titration medium was removed from the cells and 50 µL of diluted virus was added per well (four replicates per dilution). The plates were incubated at 37 °C with 5% CO_2_ for 45 min. After adding 150 µL titration medium containing 5% FCS, cells were incubated for 14 d at 37 °C with 5% CO_2_. Cells were then fixed with 4% paraformaldehyde (1.176.201.000, Morphisto, Offenbach am Main, Germany) for 24 h, removed from the BSL-4 laboratory, and permeabilized for 30 min with 0.1% Triton^®^ X-100 (3051.3, Carl Roth, Karlsruhe, Germany) in PBS. Cells were washed with 0.1% Tween^®^-20 (9127.1, Carl Roth) in PBS (PBST) and blocked for 30 min with 1% Albumin Fraction V (0163.2, Carl Roth) in PBST (Blocking solution). Cells were then incubated at 4 °C overnight with primary mouse monoclonal antibodies against EBOV-NP (ABIN5506751, antibodies-online.com) or mouse IgG2a kappa light-chain isotype control antibodies (NB600-986, Novus Biologicals, Centennial, CO, USA), both diluted 1:1000 in blocking solution. After extensive washing, cells were incubated for 1 h with goat anti-mouse IgG H&L Alexa Fluor^®^ 488 (115-545-003, Dianova) as a secondary antibody diluted 1:1000 in blocking solution. Liquid was removed from the plates and wells were examined for EBOV-infected cells using a fluorescence microscope (Evos™ FL, Life Technologies, Carlsbad, CA, USA). The endpoint dilution of positive wells was used to calculate the TCID_50_/mL using the method of Spearman and Kärber [68].

### 2.5. Microscopy

Cytopathic effects (CPE) of EBOV-infected cells were assessed using a transmitted light microscope (EVOS™ XL Core, Life Technologies, Carlsbad, CA, USA). EBOV-GFP-infected cells or EBOV-infected cells after staining were visualized using a fluorescence microscope (Evos™ FL). NPC1 receptor expression levels after infection with filoviruses were measured using confocal microcopy as described before [35]. To investigate EBOV-NP expression with confocal microscopy, antibodies were used as described above. For actin filament staining, a 100 nM solution of Acti-stain 555 Phalloidin (PHDH1-A, Cytoskeleton) was used for 30 min and samples were mounted in ddH_2_O.

### 2.6. Viral Genome Analysis

To build dual-indexed libraries for Illumina sequencing from the initial virus (EBOV_initial) and from supernatant of MoLu Prim_EBOV 150 dpi (EBOV_150dpi), we performed DNase treatment using TURBO DNA-free™ Kit (Ambion, Austin, TX, USA), cleaned up the reactions with RNA Clean & Concentrator Kit (Zymo Research, Irvine, CA, USA), converted purified RNA to cDNA using the SuperScript™ IV First-Strand Synthesis System (Invitrogen, Waltham, MA, USA), and subsequently turned it into dsDNA with NEBNEXT^®^ mRNA Second Strand Synthesis Module (New England Biolabs, Ipswich, MA, USA). DNA was purified using MagSi-NGSprep Plus Beads (Steinbrenner Laborsysteme, Wiesenbach, Germany), eluted in TET (Tris-HCl (10 mM), EDTA (1 mM), Tween20 (0.05%)), and fragmented using a Covaris S220 Focused-ultrasonicator with settings to generate 400 bp fragments (intensity = 4, duty cycle = 10%, cycles per burst = 200, treatment time = 55 s, temperature = 7 °C). Library preparation was performed with NEBNext^®^ Ultra™ II DNA Library Prep Kit for Illumina^®^ (New England Biolabs), and dual-indexes were added using NEBNext^®^ Multiplex Oligos for Illumina^®^ (New England Biolabs). Dual-indexed libraries were quantified using KAPA Library Quantification Illumina Universal Kit (Roche, Basel, Switzerland).

To enrich EBOV RNA, we followed the myBaits Hybridization Capture for Targeted NGS protocol (Version 4.01) using custom-made RNA baits (120 nucleotides long, 2-fold tiling; Arbor Biosciences, Ann Arbor, MI, USA) that cover representative genomes of *Zaire ebolavirus* (KC242801), *Sudan ebolavirus* (KC242783), *Reston ebolavirus* (NC_004161), *Taï Forest ebolavirus* (NC_014372), *Bundibugyo ebolavirus* (KC545395), and *Marburg marburgvirus* (FJ750956). Only a fourth of the recommended bait input volume was used. We prepared separate capture reactions for the two EBOV samples, and performed two 24 h long rounds of hybridization capture at a temperature of 65 °C. After both rounds of capture, capture products were amplified using the KAPA HiFi HotStart ReadyMix and Illumina adapter-specific primers, quantified using the KAPA Library Quantification Illumina Universal Kit, and cleaned up using MinElute PCR Purification Kit. The second-round product was quantified and diluted to 4 nM for sequencing on an Illumina MiSeq platform (EBOV_initial), and to 1 nM for the Illumina iSeq (EBOV_150dpi).

EBOV_initial capture product was sequenced on an Illumina MiSeq platform (V3 chemistry, 2 × 300 bp reads) for a total of 677,140 unfiltered paired reads. EBOV_150dpi was sequenced on an Illumina^®^ iSeq platform using iSeq 100 i1 Reagents (2 × 150-cycle) for a total of 1,585,308 unfiltered paired reads. Sequencing reads were filtered (adapter removal and quality filtering) with Trimmomatic [69] (settings: LEADING:30 TRAILING:30 SLIDINGWINDOW:4:30 MINLEN:40). Read pairs were merged using ClipAndMerge [70], and merged, unmerged, and unpaired reads for each sample were combined into a single file, which was mapped to a *Zaire ebolavirus* Makona strain (MG572232) using BWA-MEM [71]. For EBOV_initial, 99.55% of 734,731 high quality reads were mapped to the reference, for EBOV_150dpi 99.84% of 1,896,873 high quality reads were mapped. The mapping files were sorted and duplicates were removed with the tools SortSam and MarkDuplicates from the Picard suite [72], resulting in 41,934 and 64,015 unique mapped reads, respectively. We then used Geneious Prime to assemble consensus genomes, calling bases with a minimum coverage of 20 × and a 50% majority. The consensus sequences both contained 18,956 unambiguous positions (out of 18,958 positions in the mapping reference). To compare the genomes of EBOV_initial and EBOV_150dpi, we aligned the consensus sequences using the MAFFT v7 implemented in Geneious [73] and visually inspected the alignment.

## 3. Results

### 3.1. Low EBOV Replication Rates in Most Primary Cells from M. condylurus

**Figure 1:**
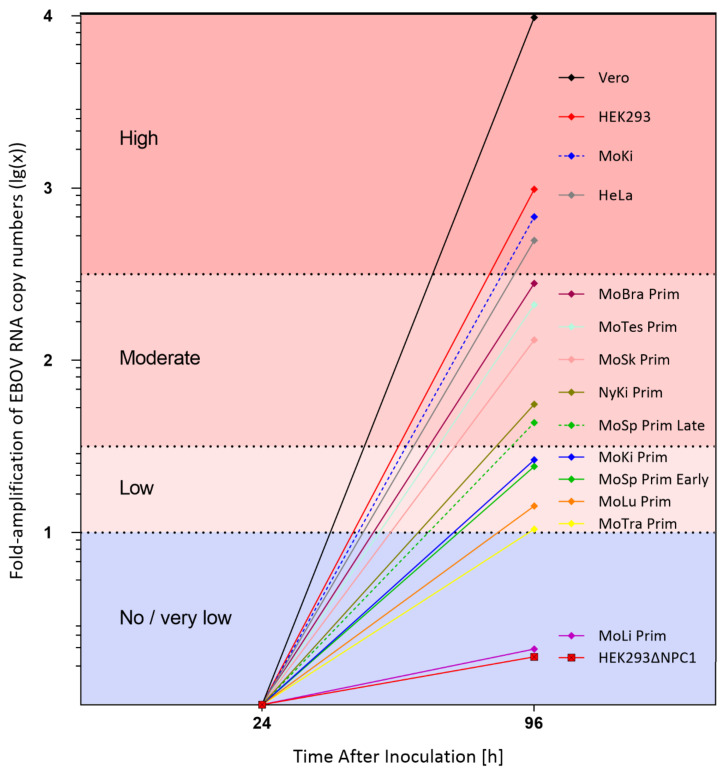
Fold-amplification of EBOV RNA copy numbers (lg(x)) between 24 and 96 hpi. Viral RNA copy numbers/mL in supernatants of infected cell isolates were determined by qRT-PCR and calculated from three replicates per experiment.

To investigate a potential correlation between EBOV replication rates and NPC1 receptor expression levels (Table 1), 15 unique cell isolates were infected with EBOV and analyzed. EBOV replication differed significantly depending on the cell isolate (Supplementary Figure S1). For comparison of virus replication rates, the fold-amplification of viral RNA copy numbers between 24 and 96 hpi was determined. Replication rates were classified as very low/no replication (lg(x) < 1.0), low (lg(x) = 1.0−1.5), moderate (lg(x) > 1.5–2.5), or high (lg(x) > 2.5) (Table 1; Figure 1).

HEK293∆NPC1 and MoLi Prim cells were refractory to EBOV infection; no virus replication was detectable within the first 96 hpi. Low virus replication rates (lg(x) = 1.02–1.42) were determined in four primary cell isolates from *M. condylurus* (MoTra Prim, MoLu Prim, MoSp Prim Early, and MoKi Prim). MoSp Prim Late, MoTes Prim, MoSk Prim, and MoBra Prim from *M. condylurus* as well as NyKi Prim from *N. noctula* showed moderate replication rates. The human and monkey cell lines (HeLa, HEK293, and Vero) and the immortalized MoKi cells had the highest replication rates, with detected amplification increases of up to approximately lg(x) = 4.

To determine the correlation between viral RNA copy numbers and viable virus, cell culture supernatants at 96 hpi from Vero, MoKi, MoBra Prim, MoTra Prim, MoLu Prim, HEK293∆NPC1, and MoLi Prim cells were titrated (Supplementary Figure S2). Overall, infectious virus titers were congruent with a virus/RNA ratio between about 1:15 (MoTra Prim) and 1:2000 (MoBra Prim). No infectious virus could be detected in supernatants of HEK293∆NPC1 and MoLi Prim cells.

### 3.2. Cytopathic Effects (CPE) in EBOV-Infected Cell Isolates

**Figure 2:**
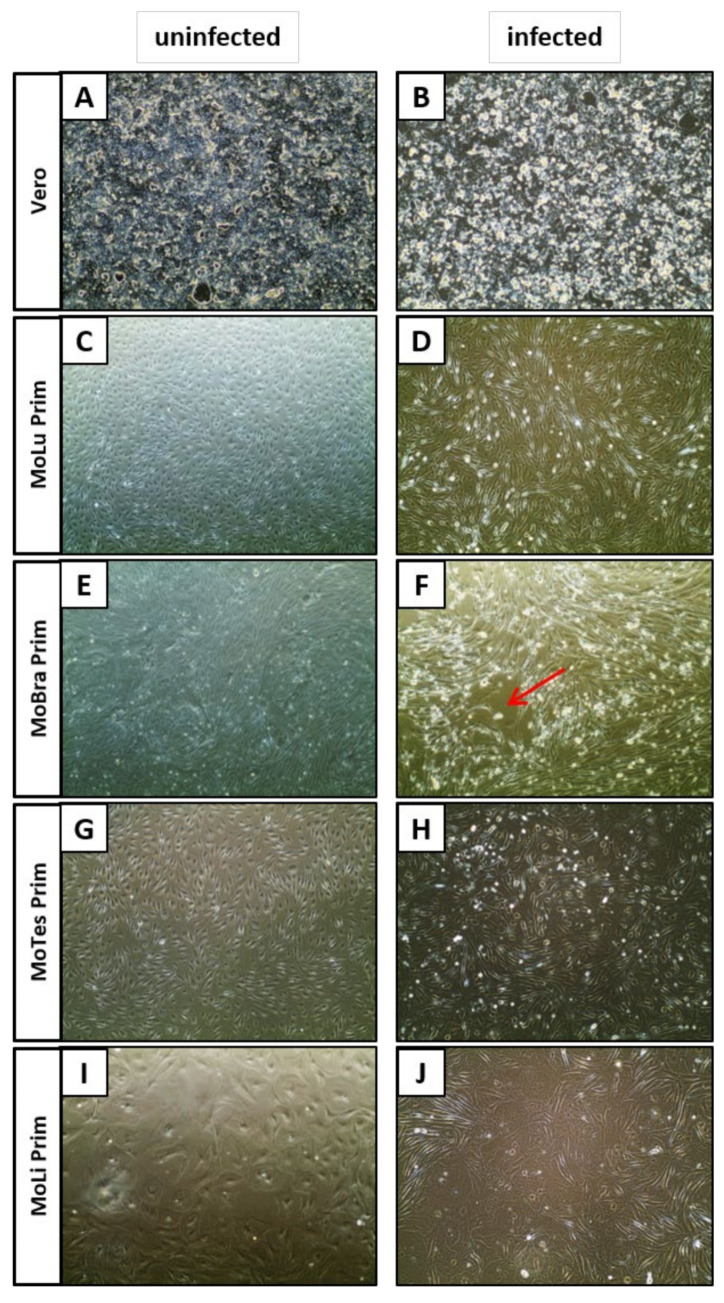
Cytopathogenicity in EBOV-infected cells. Phase contrast microscopy of uninfected cells (**A**,**C**,**E**,**G**,**I**) and EBOV-infected cells 16 dpi (**B**,**D**,**F**,**H**,**J**). CPE with enlarged cells (**B**), enhanced cell division (**D**,**H**,**J**), or plaques ((**F**), red arrow). Magnification: 10×.

EBOV-infected cells were observed out to 16 dpi for signs of CPE. Primary cell isolates from *M. condylurus* showed no (MoTra Prim and MoKi Prim) or little (MoBra Prim) CPE, whereas Vero cells became enlarged (Figure 2B), HEK293 and HeLa cells detached from the cell culture flask 7 and 10 dpi, respectively. In MoBra Prim (Figure 2F), MoSp Prim Late, and NyKi Prim cell cultures, 20–40% of the monolayer showed extensive CPE and plaques became visible, whereby 40–80% of the monolayer was still intact. Interestingly, MoLu Prim, MoTes Prim, MoLi Prim (Figure 2D,H,J), MoSp Prim Early, and MoSk Prim cells displayed enhanced cell division rates without CPE and appeared healthier compared to uninfected cells.

### 3.3. NPC1 Receptor Expression Levels Are Upregulated in Different M. condylurus Cell Isolates Following EBOV Infection

To determine how EBOV infection might influence the NPC1 receptor expression levels, we selected cell isolates from *M. condylurus* with different NPC1 basic expression levels (MoLu Prim, MoBra Prim, and MoKi) and assessed the changes of NPC1 receptor expression levels 22 days post-infection with EBOV using confocal microscopy (Supplementary Figure S3). Infection with EBOV led to a strong upregulation of NPC1 receptor expression levels in comparison to uninfected cells in all tested cell isolates and homogeneously in all cells (Supplementary Figure S3B,D,F,H). After infection, cells with low (MoLu Prim) or moderate basic expression levels (MoBra Prim and MoKi) (Table 1), high NPC1 receptor expression levels were shown. To determine whether upregulation of NPC1 is a unique response to EBOV infection, we assessed NPC1 expression after exposure of MoKi cells to a distantly related filovirus (MARV). Interestingly, MARV infection led to an even higher upregulation of NPC1 receptor expression levels than EBOV infection did (Supplementary Figure S3H).

### 3.4. EBOV Persistently Infects Primary Cells from M. condylurus

To investigate whether EBOV might establish a persistent infection in *M. condylurus* primary cells, EBOV-infected primary cells derived from lung, brain, and testicles (MoLu Prim, MoBra Prim, and MoTes Prim) were regularly passaged and visually monitored for cytopathic changes and cell death. MoLu Prim cells infected with EBOV showed division rates comparable to uninfected cells, consistently reaching 100% confluency within 7 d, and were passaged weekly until 150 dpi (Supplementary Figure S4A). Cell viability consistently ranged from 95 to 100%. EBOV-infected MoBra Prim and MoTes Prim cells were cultivated until 74 dpi with weekly changes of cell culture medium. They were only passaged twice due to their low cell division rates, while not reaching confluency (Supplementary Figure S4B,C). Cell viability ranged between 80 and 85% for both cell cultures at the time of passaging. On day 67 post-infection, MoBra Prim and MoTes Prim cells started to lose adherence and on day 74 all cells were completely detached.

**Figure 3:**
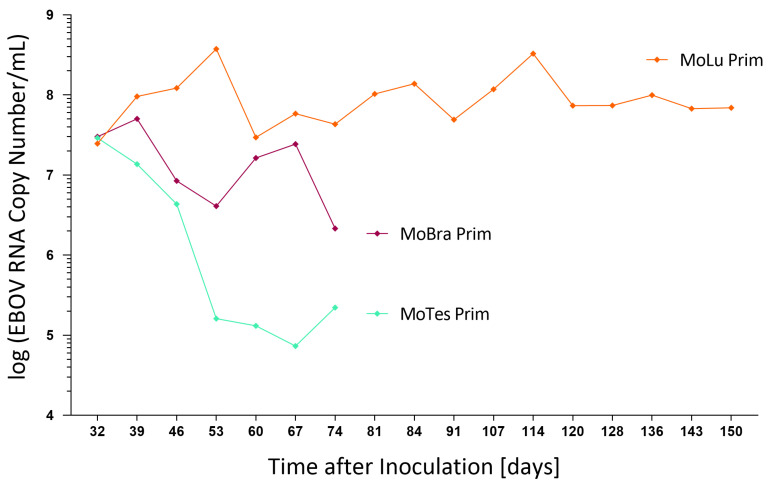
EBOV replication kinetics in long-term infected *M. condylurus* cells. Viral RNA copy numbers/mL in supernatants of infected cell isolates were determined by qRT-PCR for 74 (purple, cyan) and 150 (orange) dpi.

**Figure 4:**
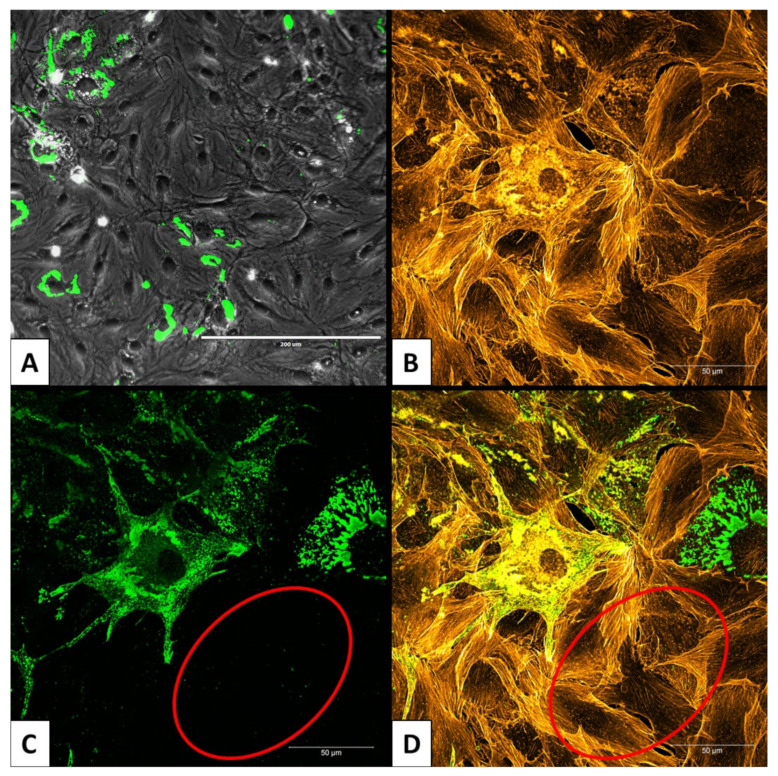
Persistently infected MoLu Prim_EBOV cells. Overview of persistently infected MoLu Prim_EBOV cells 143 dpi; scale bar: 200 µm (**A**). Enlargement of MoLu Prim_EBOV cells; scale bar: 50 µm. Stained actin filaments (**B**); stained EBOV-NP (**C**). Overlay B and C (**D**). Area with uninfected cells (red ellipse), EBOV-NP (green), actin filaments (orange).

Similar amounts of viral RNA, between 2.5 and 3.0 × 10^7^ copies/mL in supernatants, were detected for all three cell isolates at 32 dpi (Figure 3). Continuously high RNA titers, with up to 3 × 10^8^ copies/mL, were detected in MoLu Prim cells out to 150 dpi. Viral RNA titers in MoBra Prim and MoTes Prim cells dropped to 2 × 10^6^ and 2 × 10^5^ copies/mL, respectively, on day 74 post-infection. For the duration of the 150 days in culture, only a small proportion of MoLu Prim cells were persistently infected (Figure 4A), surrounded by uninfected cells (Figure 4C,D), and the cultures tolerated high levels of EBOV replication with unchanged cell division rates. For subsequent experiments, this persistently infected cell isolate was termed “MoLu Prim_EBOV”.

### 3.5. EBOV Replicates without Selective Pressure for Virus Mutations in M. condylurus Primary Lung Cells

To investigate whether EBOV might accumulate mutations due to selective pressure after long-term replication on *M. condylurus* primary cells, we compared the full viral genome sequences of EBOV used for the initial infection to EBOV from the supernatant of MoLu Prim_EBOV on day 150 post-infection. Only two changes were detected: on day 150, EBOV had one deletion in the 5′UTR in position 10 and a nucleotide change from T to G in genome position 10,590 in the VP24 CDS.

The release of EBOV from infected cells increased from 6 × 10^3^ TCID_50_/mL 96 hpi (MoLu Prim) after 21 passages to 6 × 10^5^ TCID_50_/mL 150 dpi (MoLu Prim_EBOV) (Supplementary Figure S2).

### 3.6. EBOV Infection Slowly Spreads in the Monolayer of Lung and Liver Primary Cells

**Figure 5:**
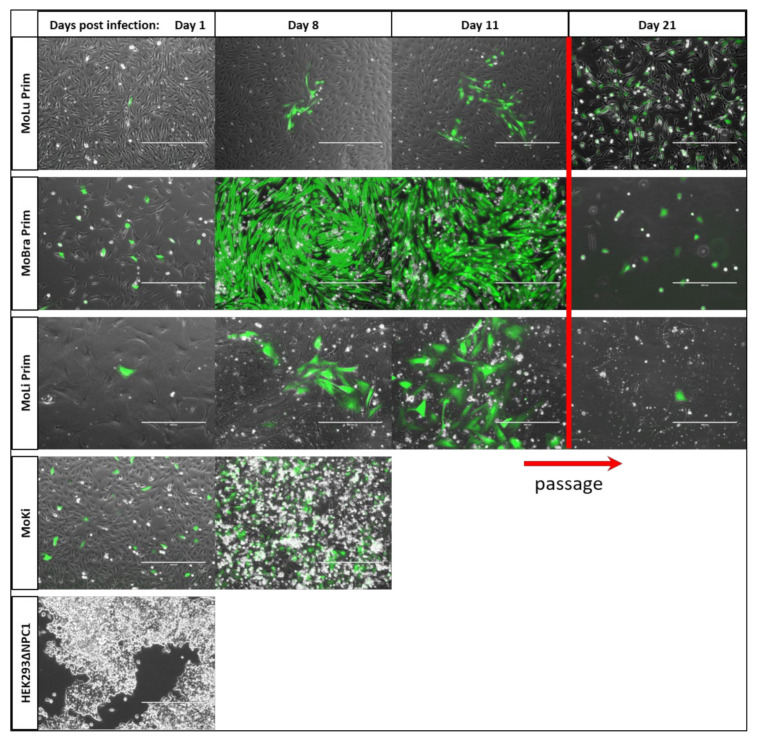
EBOV-GFP infections of different cells. Fluorescence microscopy of cell isolates infected with EBOV-GFP (green) for 21 dpi. Different number of infected cells in different cell isolates: no infected cells (HEK293∆NPC1); few infected cells (MoLu Prim, MoLi Prim); high ratio of infected cells (MoKi); very high ratio of infected cells (MoBra Prim). First passage of infected cells on day 19 (red bar). Scale bar: 400 µm.

To investigate the number of infected cells in the monolayer and the dynamics of virus spreading in different cell isolates, selected primary cells from *M. condylurus* and HEK293∆NPC1 as control were cultured and infected with EBOV-GFP (Figure 5) and monitored over 21 days. The percentage of infected cells in the monolayer differed depending on the cell isolate. MoKi cells showed the highest percentage of infected cells on day 1 post-infection and detached completely on day 11. EBOV-GFP spread slowly in the monolayer of MoLi Prim and MoLu Prim cell cultures, from single isolated infected cells (day 1), to small foci of infected cells (day 11) surrounded by many uninfected cells (Figure 5). In contrast, 100% of MoBra Prim cells were infected as early as day 8 post-infection.

MoLu Prim, MoBra Prim, and MoLi Prim cells were passaged on day 19 post-infection (Figure 5, red bar). MoBra Prim and MoLi Prim cells showed limited cell division and did not reach confluency after passaging. EBOV-GFP-infected MoLu Prim cells showed cell division rates comparable to uninfected MoLu Prim cells, reaching 100% confluency weekly and were passaged until day 62 post-infection. Cell viability was determined to be 95–100% at the time of passaging. As with EBOV, EBOV-GFP also established a persistent infection in primary lung cells from *M. condylurus*. MoLu Prim cells persistently infected with EBOV-GFP are henceforth referred to as “MoLu Prim_EBOV-GFP”. The percentage of EBOV-GFP-infected MoLu Prim cells in the monolayer on day 11 (Figure 5) was similar to the proportion in MoLu Prim_EBOV-GFP on day 62 post-infection (Supplementary Figure S5), or in MoLu Prim_EBOV on day 143 post-infection (Figure 4). As expected, there was no detectable virus replication in HEK293∆NPC1 cells until day 4, when they detached from the plate.

## 4. Discussion

Maintenance of a virus within its natural reservoir requires its persistence in the animal population and/or in individual animals, its tolerance by the host, and its replication to high enough levels to allow for transmission to naïve animals. Our results from investigating replication kinetics, tolerance, and persistence of EBOV in primary cells from *M. condylurus* provide further evidence that this microbat species may be important in the maintenance of ebolaviruses and be a likely reservoir host.

The integral membrane protein NPC1 has been described as a key component in the filoviral entry process [28,31,74,75], shown to be essential for EBOV entry [28,29,30] and MARV susceptibility [31]. While the absence of the required NPC1 receptor, as demonstrated in NPC1-knockout HEK293 cells, reliably prevents EBOV infection, NPC1 receptor expression levels correlated with the amounts of EBOV released in the supernatant in the majority of the tested cell isolates. Consequently, primary cell isolates with particularly low NPC1 receptor expression levels (MoTra Prim, MoLu Prim, MoSp Prim Early, and MoKi Prim cells) supported only low EBOV replication rates. In contrast, cells with particularly high NPC1 receptor expression levels (HeLa, HEK293, and Vero) correlated with very high virus replication rates. Comparing primary spleen cells at passage 5 (MoSp Prim Early), which have low NPC1 receptor expression levels, to the same spleen cells at passage 29 (MoSp Prim Late), which have increased NPC1 expression levels, the same correlation was observed with low and high EBOV replication rates, respectively. In two cell isolates, this correlation was not observed; MoSk Prim and NyKi Prim cells revealed moderate EBOV replication rates, but low or high NPC1 receptor expression, respectively. These results underline that the NPC1 receptor is a key component that influences tropism and replications kinetics, but not the only host cell determinant for EBOV replication. Availability of attachment factors and different efficiency of viral replication, transcription, translation, assembly, and budding of virus progeny may also influence EBOV replication rates in different cell types. In addition, the percentage of infected cells in a monolayer influences the amount of detectable viral RNA in cell culture supernatants. Additionally, the amount of virus entering cells of different cell isolates during infection may be different and would influence calculations, so that the discussed replication rates can only serve as an approximation. Future NPC1 knockdown or knockout experiments for selected cell types could confirm the correlation between NPC1 expression and EBOV replication efficiency.

Nearly all human cell types tested, and a very broad range of other mammalian cells are susceptible to EBOV infection. Only a few cell types have been described to be refractory to EBOV infection. Those that are known to be refractory are cells with D502F NPC1 polymorphism from *Eidolon helvum* fruit bats [34], cells from patients with Niemann–Pick C1 disease [31], cells of lymphoid origin [27,76,77] including human B-, T-, and NK cells, murine lymphoid cell lines or mosquito cells [78]. Despite the moderate NPC1 receptor expression, no distinct EBOV replication could be observed in supernatants of MoLi Prim cells within the first 4 days of infection. Using EBOV-GFP, we demonstrated that infection of MoLi Prim cells is inefficient, but cells were not refractory, with only few cells in the monolayer being infected, suggesting a possible impaired virus entry by insufficiently expressed cellular attachment factors. Several EBOV-infected MoLi Prim cells in the monolayer developed into small clusters of infected foci, indicating a very inefficient infection of cells, followed by strong replication, but no detectable release of infectious virus particles. Instead, infection of neighboring cells seemed to result exclusively from cell-to-cell transmission, which has not been described for filoviruses. Virus components might be transmitted into neighboring cells via intercellular pores, as described for measles virus in human airway primary cells [79].

We also observed that EBOV (and also MARV) upregulate NPC1 to very high levels, regardless of the original cellular expression level. Markedly increased NPC1 receptor expression levels were detected in all cells within a well 22 days after EBOV or MARV infection in MoLu Prim, MoBra Prim, and MoKi cells, regardless of individual cells being infected or not. Although only a small number of MoLu Prim cells became infected, the effect of strong NPC1 upregulation was observed for all cells in a given culture. We hypothesize that a thus far unknown soluble factor(s), secreted from infected cells into the supernatant, is causing the NPC1 expression level changes. Future experiments have to reveal the nature of this phenomenon and how fast the effect of receptor upregulation can be detected after infection with filoviruses. Interestingly, EBOV infection was not increased following NPC1 upregulation, indicating a changing importance of NPC1 during the course of infection. The described correlation between low NPC1 receptor expression levels and EBOV replication rates may have a higher impact in the early course of infection, while later, after general upregulation of NPC1 expression levels, other factors might predominantly influence the efficiency of virus replication in different cell isolates.

In humans, high levels of viral replication lead to lysis and necrosis in cells of many organs, including the liver [80], which strongly contributes to the pathogenesis of Ebola virus disease. Interestingly, all primary cells from *M. condylurus* produced only low or moderate EBOV replication rates compared to human and monkey cells. Translating the low EBOV replication in most of the primary cell isolates from *M. condylurus* to the potential outcome in the host, one can assume comparable low virus replication rates in the corresponding organs, which could contribute to asymptomatic infections in these microbats. Previous studies of EBOV entry and infection processes in bat cells, predominantly performed in immortalized cell lines [34,77,81,82,83,84,85,86,87,88] have ascertained that cells from a wide variety of bat species support filovirus replication in vitro leading generally to high virus titers [81,84]. Primary and corresponding immortalized cells of the same organ source (MoKi Prim and MoKi) revealed low and high virus replication rates, respectively. The amplification of EBOV RNA was more than 600-fold higher in immortalized MoKi cell supernatant, confirming the genetic and phenotypic difference of MoKi cells from their in vivo counterparts, while primary cells likely maintain many of the important markers and functions seen in vivo [89,90,91].

Human cells (HEK293 and HeLa) and a primary cell isolate from the European microbat *N. noctula* (NyKi Prim, unlikely to be a reservoir host of EBOV) showed strong CPE with cell death after infection with EBOV, while the first two lost their adherence on days 7 and 10 post-infection, respectively. Plaques and destruction of the cell monolayer after infection with EBOV in *M. condylurus* cell isolates could only be observed for spontaneously immortalized MoSp Prim Late and for MoBra Prim cells, whereby for the latter, only 20% of the monolayer was affected 16 dpi. All seven other primary cell isolates from *M. condylurus* did not develop any CPE (MoTra Prim and MoKi Prim) or showed increased cell division rates (MoLu Prim, MoTes Prim, MoLi Prim, MoSp Prim Early, and MoSk Prim) compared to uninfected control cells. Mandl et al. hypothesized that viruses are more likely to be cytopathic in non-natural hosts, while viral infections are better tolerated by reservoir hosts even when viral loads are high [92]. In this context, tolerance is defined as the ability to limit the health impact caused by a pathogen [93,94], so that health and fitness are maintained, despite virus replication. In our study, tolerance to EBOV could only be observed for primary cells from *M. condylurus* and no noticeable cell damage or destruction could be detected in most of these cells. Although higher EBOV RNA titers were observed for some primary cell isolates from *M. condylurus* (MoSk Prim or MoTes Prim, 96 hpi), cells showed high viability and could be passaged several times. Remarkably, MoLu Prim_EBOV cells tolerated high amounts of virus in the supernatant and intracellularly for 150 days without any signs of cytopathology. Tolerance to EBOV at the cellular level suggest a similar outcome in the corresponding organs in vivo, which would result in a lower degree of cell damage and therefore contribute to asymptomatic infections in *M. condylurus*. The importance of tolerance for asymptomatic infections of this potential reservoir host has to be determined in further in vivo experiments.

To investigate the intrinsic ability of *M. condylurus* primary cells to support EBOV persistence, cells were passaged and cultivated for several weeks post-infection. In contrast to primate cells, all examined primary cells from *M. condylurus* showed characteristics that support tolerance to EBOV and establishment of persistent infections. Only infected lung primary cells reached confluency, were passaged weekly, and showed a permanent persistence of EBOV for five months, after which the experiment was stopped. The virus and the host cells seemed to be in equilibrium, such that virus replication and high virus titers were permanently tolerated without cell damage or impairment of cell division and viability. The persistence of EBOV in MoLu Prim cells was recapitulated with EBOV-GFP in an independent experiment, indicating a reliable outcome for this cell isolate. Conversely, no permanent persistence could be observed in *M. condylurus* primary cells derived from immune privileged organs (brain and testicles), in which EBOV was reported to persist in humans [59,60,61,95]. However, the special characteristics of these organs such as the blood–brain and blood–testis barriers, or the suppression of proinflammatory immune responses, cannot be simulated in cell culture, so that the validity of our results concerning virus persistence in these organs of the potential reservoir host is rather limited. During the persistent EBOV infection in MoLu Prim cells, only a small proportion of cells in the monolayer became infected, although NPC1 receptor expression levels were already markedly increased. A relatively high virus titer of 6 × 10^5^ TCID_50_/mL on day 150 post-infection resulted from efficient virus replication in the relatively few infected cells, indicating that high virus titers in the supernatant do not necessarily reflect the overall permissiveness of a cell culture. For the establishment of virus persistence, a repression of virus replication in at least some infected cells or a slow spread from cell-to-cell are potential mechanisms [37]. Although the specific mechanism of EBOV persistence in vitro and in vivo cannot be determined based on our data, they indicate virus replication occurs in individual cells of certain organs only. While the virus spreads slowly from cell-to-cell, the majority of cells remain uninfected and can presumably fulfil their specific functions in the organ, which may also facilitate an asymptomatic infection in the reservoir host. McCarthy et al. showed, that respiratory syncytial virus (RSV) may persist for months in lungs and airways following acute infection, although the mechanisms of persistence are poorly understood [96]. The recent discovery of RNA from a previously unknown filovirus in lungs of *Rousettus* and *Eonycteris* fruit bats in China [97,98], repeated detection of Bombali virus RNA in lungs of *M. condylurus* bats [24,26] and repeated establishment of EBOV persistence in lung primary cells from *M. condylurus* indicates that ebolaviruses may persist in vivo in lungs of this bat species. Detection of EBOV in lungs of *M. condylurus* after experimental infection implies that respiratory or oral spread of infection could occur in the confined spaces of bats roosts [22]. Future experiments may reveal mechanisms that allow EBOV persistence in *M. condylurus* primary cells and whether ebolaviruses can persist in vivo in certain organs of this microbat species.

Another mechanism contributing to the establishment of virus persistence is the accumulation of defective interfering (DI) particles, which may dampen virus replication [64,99,100,101,102,103]. Ten undiluted passages of EBOV with high MOI (>1) resulted in production of DI particles and persistently infected Vero cells [104]. In contrast, in our experiment the amount of virus used to infect MoLu Prim cells was low and the amount of infectious virus in the supernatant increased over time. Therefore, the establishment of EBOV persistence in lung primary cells of *M. condylurus* was not enforced through accumulation of DI particles as described by Calain et al. but seemed to be facilitated by specific characteristics of the cells. Additionally, the selection of virus variants with reduced cytopathogenicity may be a mechanism during the establishment of persistent infections [37,64,105,106,107,108]. As MoLu Prim cells tolerated EBOV in several experiments directly after infection, such selection processes seemed to be unlikely. Comparing the full EBOV genome sequences of the inoculum and in the supernatant of MoLu Prim_EBOV cells on day 150, only one nucleotide change from T to G in position 10,590 in the VP24 CDS was detected, resulting in an amino acid change from Arginine to Lysine (both are basic amino acids with similar characteristics). Therefore, no selective pressure for virus mutation and selection of virus variants was observed.

Repeated establishment of persistent EBOV infections in primary cells from *M. condylurus* might reflect the intrinsic ability, that ebolaviruses may persist and be permanently maintained in this bat species and in the bat population, being an essential criterion for a natural reservoir [36,56,109]. Difficulties in isolating infectious Nipah virus (NiV) from wild-caught or experimentally infected bats may be, due to near elimination by immune mechanisms, but this virus likely persists at low levels in specific organs [110]. Additionally for MARV, long-term viral persistence in *R. aegyptiacus* bats has been indirectly observed, due to late transmissions from experimentally infected to naive contact bats 7 months post-infection [55,56]. In vivo studies will elucidate whether EBOV is also tolerated in *M. condylurus* bats and can persist without being eliminated by innate and adaptive immune responses. The potential persistence of ebolaviruses in lungs of *M. condylurus* bats with intermittent viral shedding may explain why no infectious virus has been detected in this or any other bat species, and why spillovers occur rarely.

In summary, low NPC1 receptor expression levels in most primary cells from *M. condylurus*, compared to human or monkey cell lines or cells from a European microbat, might contribute to low level virus replication rates in all tested primary cells from *M. condylurus* and might reflect a potential adaptation between EBOV and its natural reservoir. Most cells derived from *M. condylurus* showed high tolerance and no cell damage upon infection with EBOV, while cell destruction was observed in control cells. We noticed the repeated establishment of EBOV persistence in primary lung cells from *M. condylurus*, potentially reflecting the intrinsic ability of in vivo persistence in this bat species. With lower NPC1 receptor expression levels, lower EBOV replication rates, high tolerance to EBOV infections, and repeated establishment of persistent infection of primary cells from *M. condylurus* with slow virus spread in the monolayer, these in vitro experiments provide additional evidence that this bat species might develop asymptomatic infections in vivo and is a likely reservoir of ebolaviruses. To investigate the course of infection, the tissue tropism and the general role of these bats for the ecology of ebolaviruses, experimental infections of *M. condylurus* bats might be inevitable.

## Figures and Tables

**Table 1:** List of cell isolates used, origin, and fold-amplification of EBOV RNA copy numbers.

Cell Culture	Origin	Fold-Amplification of EBOV RNA Copy Numbers (lg(x))	Replication Rate [lg(x)]	
Vero	NHP Kidney	3.99	High	>2.5
HEK293	H Kidney	2.99		
MoKi	MC Kidney	2.83		
HeLa	H Cervix	2.70		
MoBra Prim	MC Brain	2.45	Moderate	>1.5–2.5
MoTes Prim	MC Testicle	2.32		
MoSk Prim	MC Skin	2.12		
NyKi Prim	NN Kidney	1.74		
MoSp Prim Late	MC Spleen	1.64		
MoKi Prim	MC Kidney	1.42	Low	1.0–1.5
MoSp Prim Early	MC Spleen	1.38		
MoLu Prim	MC Lung	1.15		
MoTra Prim	MC Trachea	1.02		
MoLi Prim	MC Liver	0.33	No/Very Low Replication	<1.0
HEK293∆NPC1	H Kidney	0.28		

For calculation, the viral RNA copy numbers between time points of 24 and 96 hpi were considered. Colors in the cell culture column indicate the NPC1 receptor expression level: no (lilac), low (light red), moderate (red), and high expression (dark red) [35]. Replication rate was classified as low (lg(x) = 1.0–1.5), moderate (lg(x) > 1.5–2.5), or high (lg(x) > 2.5). MC = *Mops condylurus*; NN = *Nyctalus noctula*; H = human; NHP = non-human primate.

## Data Availability

Not applicable.

## Supplementary Materials

The following are available online https://www.mdpi.com/article/10.3390/v13112186/s1, Figure S1: Ebola virus (EBOV) replication kinetics in different cell isolates, Figure S2: Comparison of EBOV RNA and virus titers in selected cell culture supernatants, Figure S3: Comparison of NPC1 receptor expression levels in *M. condylurus* cells after infection with EBOV and MARV using confocal microscopy, Figure S4: Long-term cultivation of EBOV-infected primary cells from *M. condylurus*, Figure S5: Persistently infected MoLu Prim_EBOV-GFP cells 62 dpi.
