# Supplementary material for: Tolerance and Persistence of Ebola Virus in Primary Cells from Mops condylurus, a Potential Ebola Virus Reservoir
Source: Viruses. 2021 Oct 29;13(11):2186. doi: 10.3390/v13112186 (PMC8622823; doi:10.3390/v13112186)
Supplement: Supplementary file 1 [file viruses-13-02186-s001.zip › viruses-1369063-supplementary.pdf]

viruses

MDPI

Supplementary Materials

Tolerance and Persistence of Ebola Virus in Primary Cells from *Mops condylurus*, a Potential Ebola Virus Reservoir

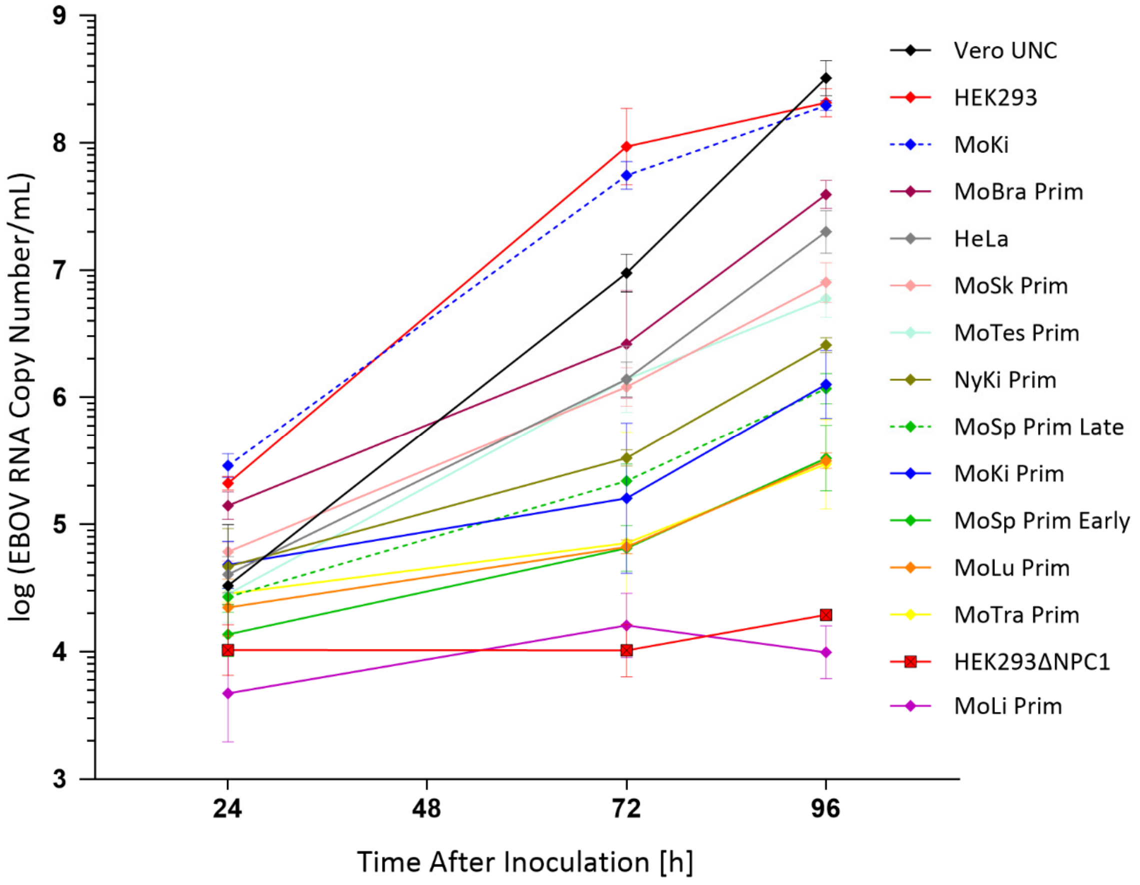

Figure S1. Ebola virus (EBOV) replication kinetics in different cell isolates. Viral RNA copy numbers/ml in supernatants of infected cell isolates were determined by qRT-PCR. Different levels of EBOV replication: No or very low virus replication (MoLi Prim, HEK293ΔNPC1), low virus replication (MoTra Prim, MoLu Prim, MoSp Prim Early, MoKi Prim), moderate virus replication (MoSp Prim Late, NyKi Prim, MoTes Prim, MoSk Prim, MoBra Prim) and high virus replication (HeLa, MoKi, HEK293, Vero). The individual viral RNA copy numbers were calculated from three replicates per experiment.

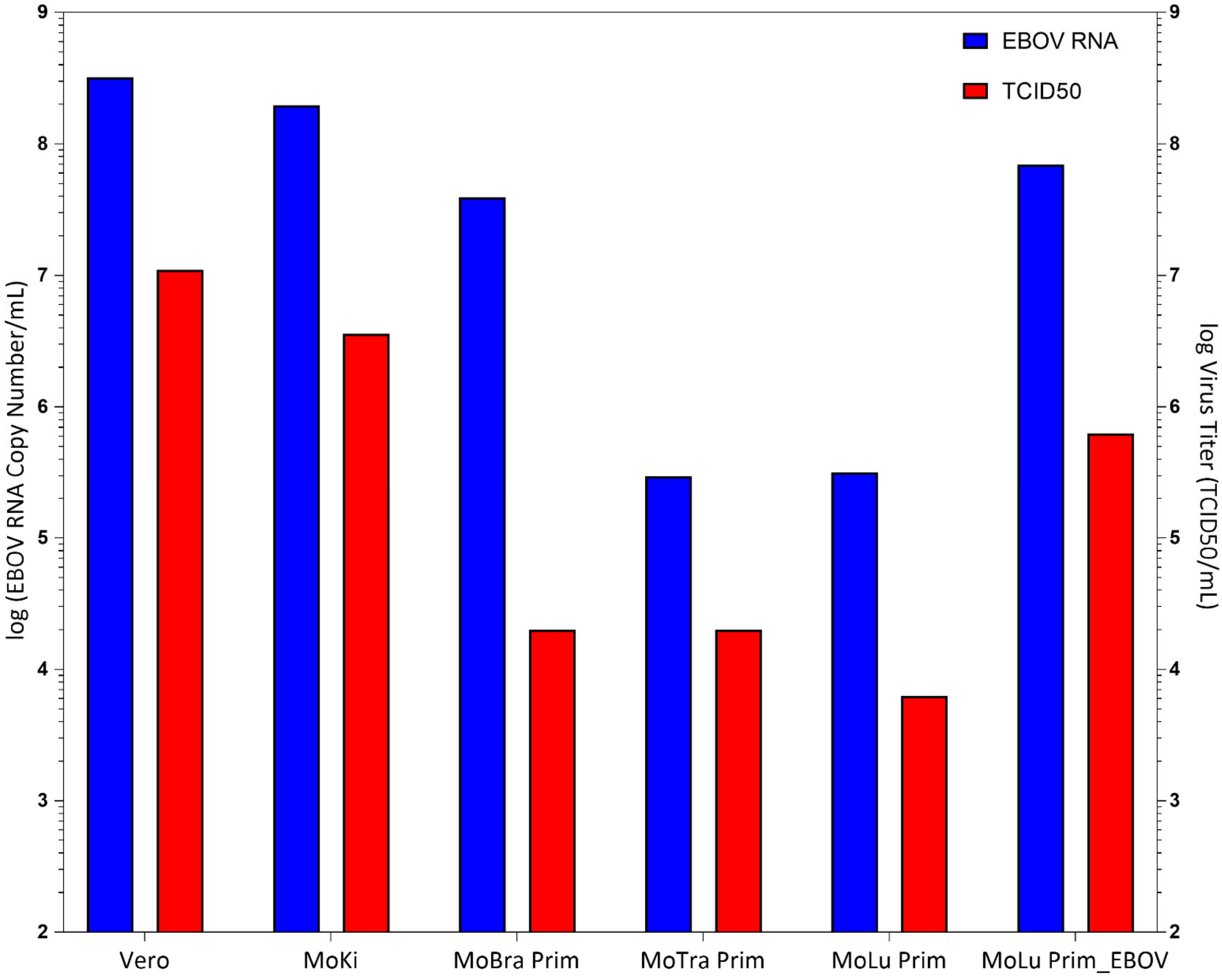

Figure S2. Comparison of EBOV RNA and virus titers in selected cell culture supernatants. Viral RNA copy numbers/ml (blue, left y-axis) and virus titers as $TCID_{50}$/mL (red, right y-axis) in supernatants of infected cell isolates. Supernatant from Vero, MoKi, MoBra Prim, MoTra Prim und MoLu Prim 96 h post infection. Supernatant from MoLu Prim_EBOV 150 days post initial infection.

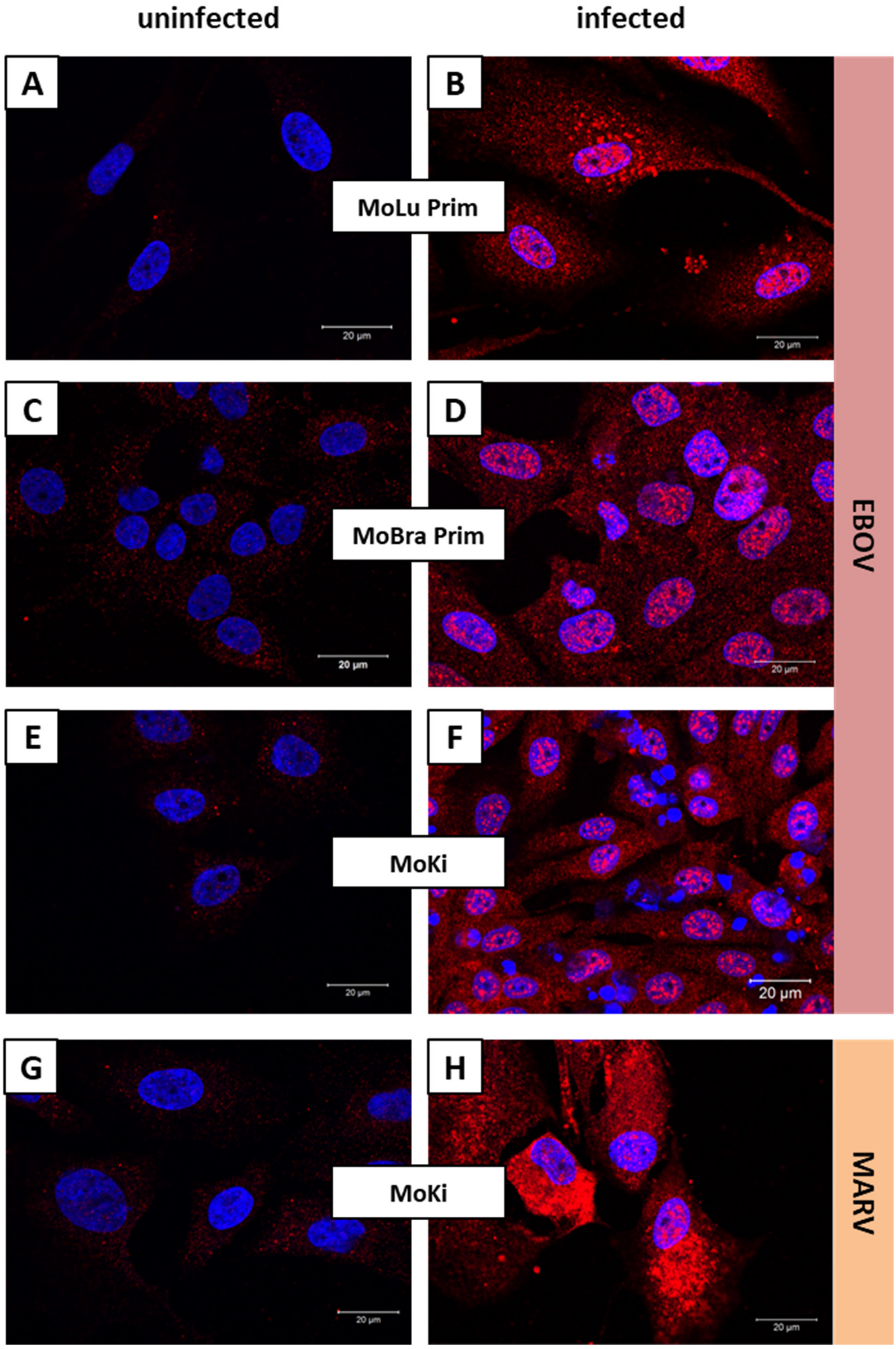

Figure S3. Comparison of NPC1 receptor expression levels in *M. condylurus* cells after infection with EBOV and MARV using confocal microscopy; scale bar: 20 µm. Stained NPC1 receptor (red), cell nuclei (blue). Left: uninfected cells (A: MoLu Prim, C: MoBra Prim, E und G: MoKi); Right: EBOV-infected cells (B: MoLu Prim, D: MoBra Prim, F: MoKi) and MARV-infected cells (H: MoKi) with high NPC1 receptor expression levels 22 days post-infection.

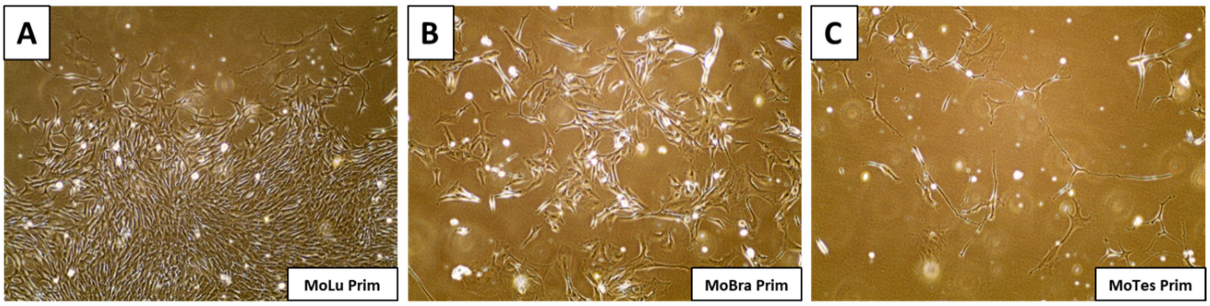

Figure S4. Long-term cultivation of EBOV-infected primary cells from *M. condylurus.* Lung (A), brain (B) and testicle (C) primary cells from *M.* *condylurus* 37 days post infection with EBOV. A: MoLu Prim cells showing high cell division rates and areas with 100 % confluence; B: Slow-growing MoBra Prim cells with low cell density; C: MoTes Prim cells with hardly any detectable cell divisions. Phase contrast microscopy: 10×.

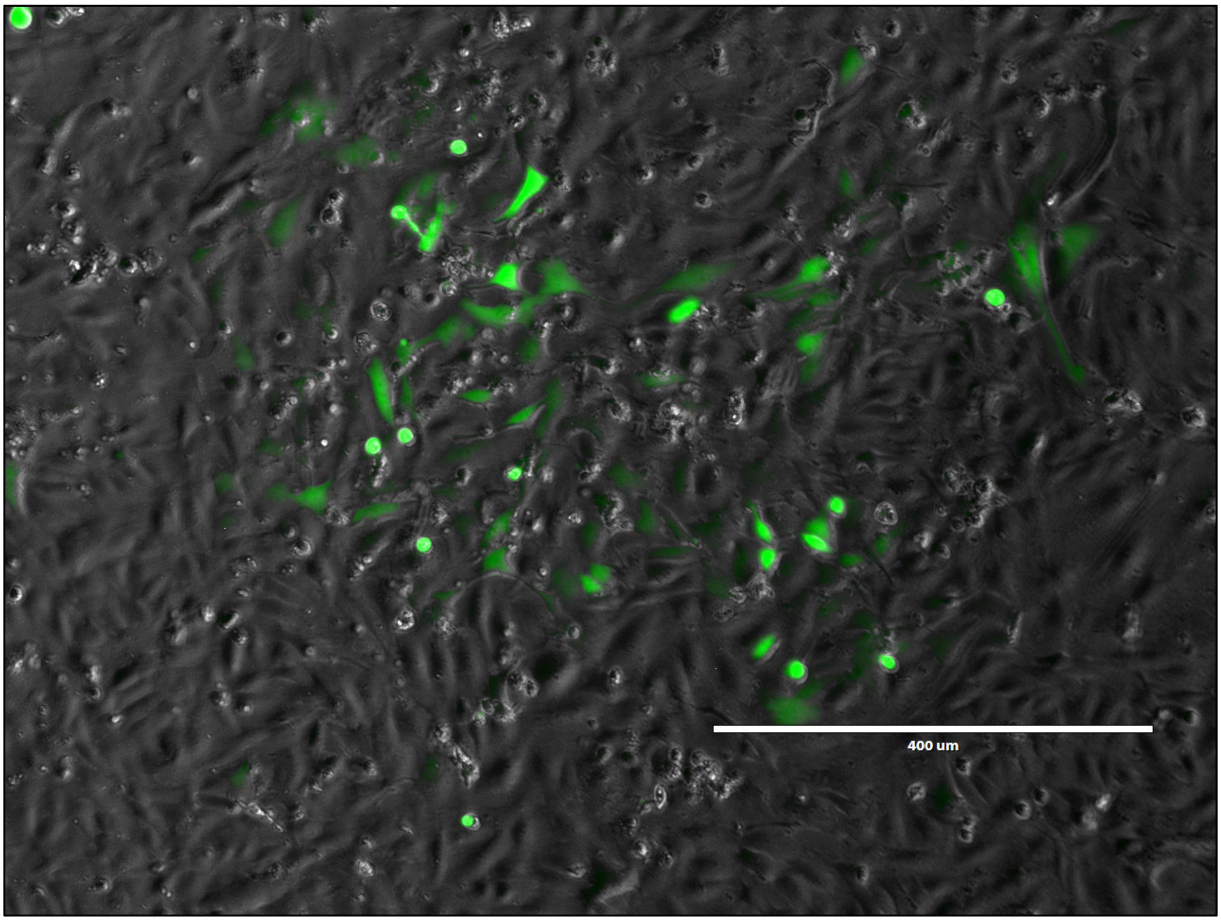

Figure S5. Persistently infected MoLu Prim_EBOV-GFP cells 62 days post initial infection; scale bar: 400 µm. GFP (green) was produced in infected cells**.** Only a small proportion of cells in the monolayer was infected 62 days post initial infection.
